# Assessment and Classification of Service Learning: A Case Study of CS/EE Students

**DOI:** 10.1155/2014/183732

**Published:** 2014-09-11

**Authors:** Han-Ying Kao, Yu-Tseng Wang, Chia-Hui Huang, Pao-Lien Lai, Jen-Yeu Chen

**Affiliations:** ^1^Department of Computer Science and Information Engineering, National Dong Hwa University, No. 1, Section 2, Da-Hsueh road, Shoufeng, Hualien 97401, Taiwan; ^2^Department of Business Administration, National Taipei University of Business, No. 321, Section 1, Jinan road, Zhongzheng District, Taipei City 100, Taiwan; ^3^Department of Electrical Engineering, National Dong Hwa University, No. 1, Section 2, Da-Hsueh road, Shoufeng, Hualien 97401, Taiwan

## Abstract

This study investigates the undergraduate students in computer science/electric engineering (CS/EE) in Taiwan to measure their perceived benefits from the experiences in service learning coursework. In addition, the confidence of their professional disciplines and its correlation with service learning experiences are examined. The results show that students take positive attitudes toward service learning and their perceived benefits from service learning are correlated with their confidence in professional disciplines. Furthermore, this study designs the knowledge model by Bayesian network (BN) classifiers and term frequency-inverse document frequency (TFIDF) for counseling students on the optimal choice of service learning.

## 1. Introduction

Educators in universities have recognized a fact that merely professional knowledge is not sufficient in real-world competition. In addition to professional know-how, the key success factors to a remarkable career include integrity, leadership, interpersonal skills, sympathy to others, and sense of social responsibilities. So students are motivated to provide voluntary services for the public and communities that can play a key role as connections to their social networks. Service learning is a form of experiential education in which students engage in activities that address human and community needs together with opportunities designed to promote student learning and development [1].

Service learning has a long history in the United States and has been recognized as key curriculum component in universities [2–6]. The forms of service learning include homeless shelters, collecting and handing out food, battered women shelters, hot lines, school tutors, and habitat for humanity [7]. Benefits of service learning have been mentioned in various investigations. Toncar et al. [4] developed the service learning benefit (SELEB) as a scale that measures student perceptions of service learning experiences. Werder and Strand [5] later assess the effectiveness of service learning in the public relations capstone course by measuring perceived student learning outcomes. Gallini and Moely [8] find that the service learning courses promoted interpersonal, community, and academic engagement, were academic challenging, and encouraged students continued study at the university (retention).

On the other hand, the challenges of service learning have been reported. Some people argue that it is neither the college's place nor position to require service learning for students. Faculty also faces the challenge that the community should not be treated as a laboratory. Besides it is difficult to prove that service learning experiences are related to the students' curriculum [7].

This study intends to aim at students' perception of service learning experiences. Among the researches on measuring the perceived outcomes, most investigate the students in social science disciplines while the benefits for engineering students are to be explored. This study first investigates the undergraduate students in computer science/electric engineering (CS/EE) to measure their perceived benefits from service learning coursework. Besides, the benefits of service learning and their correlation between the professional disciplines are tested. Furthermore, this study advances one further step to construct the counseling knowledge base using Bayesian networks. We analyze students' profiles and their feedback by term frequency-inverse document frequency and design the Bayesian network that provides advices to students for their optimal type of service learning.

*Background of the Knowledge Base.* Bayesian networks (BN) [9–11] are directed acyclic graphs (DAG) in which the nodes represent the variables, the arcs represent the direct causal influences between the linked variables, and the influences may be quantified by conditional probabilities. They are widely used knowledge representation and reasoning models in various domains under uncertainty. Since an expert system requires both predictive and diagnostic information, two types of reasoning are common in Bayesian networks, deduction and abduction. Deduction, or prediction, is a logical process from a hypothesis to deduce evidence where probabilistic relationships are involved. Abduction, or diagnosis, is a logical process that hypothetically explains experimental observations [11]. The naïve Bayesian network (NBN) model is named by Titterington et al. [12] for its simplicity. In a NBN model, the target variable of interest is normally defined as the root without a parent node.

Term frequency-inverse document frequency (TFIDF) [13–15] is the formal measure concerning how concentrated into relatively few documents the occurrences of a given word are. Suppose we have a collection of *N* documents. Define *f*_*ij*_ to be the frequency (number of occurrences) of term (word) *i* in document *j*. Then, the term frequency TF_*ij*_ is defined as follows:

(1) $$\begin{array}{c} {\text{TF}}_{ij}=\frac{f_{ij}}{\text{ma}{\text{x}}_{k}f_{kj}}; \end{array}$$

that is, the term frequency of term *i* in document *j* is  *f*_*ij*_  normalized by dividing it by the maximum number of occurrences of any term (perhaps excluding stop words) in the same document. Thus, the most frequent term in document *j* gets a TF of 1, and other terms get fractions as their term frequency for this document.

Suppose term *i* appears in *n*_*i*_ of the *N* documents in the collection. Then IDF_*i*_ = log_2_(*N*/*n*_*i*_). The TFIDF score for term *i* in document *j* is then defined to be TF_*ij*_ × IDF_*i*_. The terms with the highest TFIDF score are often the terms that best characterize the topic of the document. The measure TFIDF identifies words in a set of documents that are useful for determining the topic of each document [15].

The remainder of this paper is organized as follow.  Section 2 first develops the research design for the benefits of service learning.  Section 3 then addresses the statistics and hypotheses testing. The classification model of service learning is built at the second stage in Section 4. Finally we give the discussions and conclusions in the fifth section.

## 2. The Framework for Benefit Measurement

**Figure 1:**
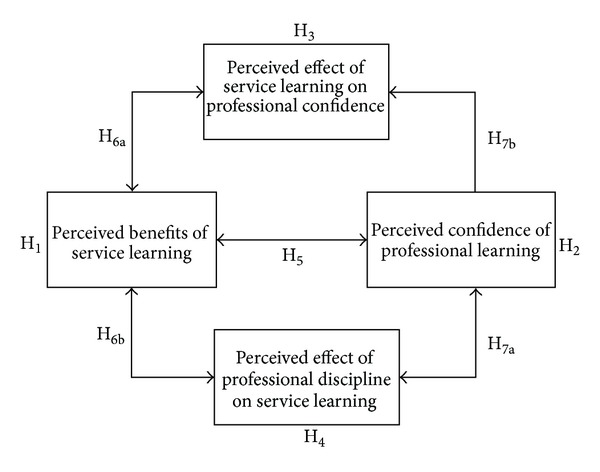
The framework for benefit measurement.

In the first stage, this study aims at the benefits of service learning and their relationships between professional capabilities. The purposes of this stage are (1) exploring the benefits of service learning course by measuring the attitudes of students in CS/EE, (2) examining students' confidence in the fields of CS/EE, (3) testing the correlation between the benefits of service learning and students' professional confidence, and (4) examining the relation between the benefits/confidence and the perceived mutual effects between service learning and professional confidence. The research framework is shown as Figure 1.

Based on the framework in Figure 1, we first test the perceived benefits of service learning and professional learning.

 H_1_: students in CS/EE perceive considerable benefits from the service learning course.
 H_2_: students in CS/EE perceive considerable confidence from professional learning.

To test the correlation between the benefits of service learning and the confidence in professional discipline, the following hypotheses are tested.

 H_3_: students perceive that their capabilities and experiences gained from service learning assist in learning the professional discipline.
 H_4_: students perceive that their capabilities in the professional discipline assist in the service learning coursework.

Besides, we test the following hypotheses on the mediating effects between the perceived benefits of two types of learning and their correlation.

 H_5_: students who perceived high benefits from service learning are likely to perceive high benefits from professional discipline (and vice versa).
 H_6a_: students who perceived high benefits from service learning are likely to perceive that service learning assists in professional learning.
 H_6b_: students who perceived high benefits from service learning are likely to perceive that professional learning assists in service learning.
 H_7a_: students who perceived high confidence of professional learning are likely to perceive that professional learning assists in service learning.
 H_7b_: students who perceived high confidence of professional learning are likely to perceive that service learning assists in professional learning.

## 3. Research Design and Hypotheses Testing

### 3.1. The Sample

We survey the attitudes of undergraduate students in CS/EE at one leading national university in Eastern Taiwan. The samples are students registered in service learning as a requisite course for two semesters during 2010–2012. In each semester they are required to provide at least 18 hours voluntary services on or off campus, under supervision of the faculty. On completion of the coursework, the students are invited to fill the questionnaires composed of 20 questions on service learning, 9 questions on professional learning, 2 questions on service experiences, and a set of questions about personal information. The total sample size is 256, including 143 in electric engineering and 113 in computer science. Finally the effective sample size is 116, among which 59 (50.86%) in electric engineering and 57 (49.14%) in computer science.

### 3.2. Questionnaire Design

The questionnaire is prepared based on [4, 5] as in Table 1. Questions in the first section construct the students' perceived benefits from service learning, and the items in the second section measure the students' perceived capabilities from professional learning. All the questions are answered with the Likert-type 7-point scale, where 1 means “strongly disagree” and 7 means “strongly agree.”

The results of Part 1 are processed by factor analysis and yield a four-factor solution. The rotated factor matrix is shown in Table 2, where all variables load significantly at least one factor. Usually factor loadings exceeding 0.5 suggest significance in practice. All factors load exceeding 0.5 except item 19 (organizational skills) and item 15 (sensitivity to the plight of others) that are slightly below 0.5. So we accept the results of factor analysis and name the factors as “*practical skills and development*,” “*critical thinking*,” “*leadership and interpersonal skills*,” and “*citizenship*,” respectively. The descriptive statistics are organized by factors as in Table 3.

### 3.3. Hypothesis Testing

To test the hypotheses H_1_ to H_4_, we take the Student's *t*-test for “mean ≥ 5” and “mean ≥ 4.5” at the 99% confidence level, respectively. From Part 1 of Table 3, most items support H_1_, except the item in factor 4 “understanding cultural and racial differences.” The responses arise likely because most students did not work in an international environment. In Part 2, 6 out of 9 items are rejected with “mean ≥ 5” but all accepted with “mean ≥ 4.5.” The results show that undergraduate students in CS/EE are fairly confident of their professional major, not at a strong level. As for the interactions between service learning and professional disciplines, both items in Part 3 reject H_3_ and H_4_, respectively.

To test H_5_, the Pearson correlation between the benefits from service learning and those from professional learning is 0.483. The correlation indicates that the benefits from two types of learning are positively correlated in a moderate manner, so H_5_ is not rejected.

For H_6a_, the correlation between perceived benefits from service learning and the first item of Part 3 is 0.677, which supports H_6a_. Similarly, the correlation between perceived benefits from service learning and the second item of Part 3 is 0.631, so H_6b_ is supported.

To test H_7a_, the correlation between perceived benefits from professional discipline and the first item of Part 3 is 0.630, and the correlation between perceived benefits from professional learning and the second item of Part 3 is 0.572. So H_7a_ and H_7b_ are both supported.

Notably there is one open question in Part 4 “please describe your experiences in service learning in words.” In next section we will describe how to analyze the responses to the open question and how to build the classification model with the personal features and the outcomes of document analysis.

## 4. Knowledge Model of Service Learning

For counseling students on service learning, this section develops the classification model by the naïve Bayesian network. In the naïve Bayesian network, the target variable (root) is students' perceived benefit level and the predicting variables are measured from the items in Part 4, including personal features and internal responses of the students.

**Figure 2:**
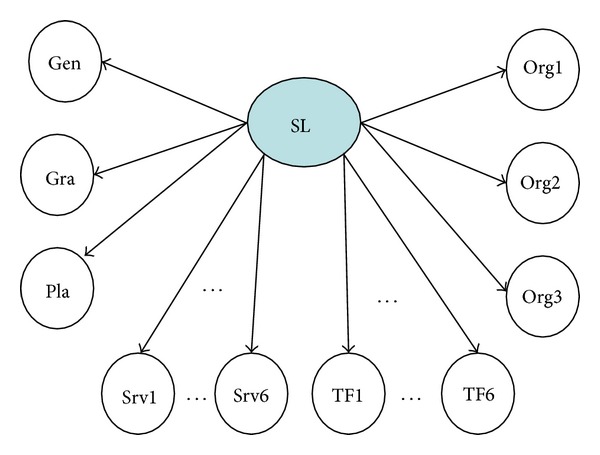
The Bayesian network on service learning benefits.

This study uses TFIDF to extract a set of words that are frequent in the students' answers. At first we select 10^2^ = 100 words. After computing the weight (importance) of the words and eliminating the “stop words” (articles, prepositions, and other auxiliary words), a compact set of 20 words were extracted. These 20 words are further factorized and analyzed, among which six terms are finally selected: “*learning*,” “*service*,” “*profession*,” “*process*,” “*document,*” and “*capability*.” The target variable and predicting variables for the Bayesian network on service learning are summarized in Table 4. The structure of the Bayesian network is depicted as Figure 2.

To estimate the parameters, this study adopts the 5-fold cross validation. The dataset is first divided randomly into five subsamples. Then in every fold, four subsets are input for learning and one subset is used for testing under various scenarios. Before learning the conditional probabilities, the continuous root variable needs to be discretized. We partition the domain of the root into 3 and 5 subranges and compare the classification accuracy by 3 and 5 states, respectively. The results of tests are summarized in Table 5.

## 5. Discussions and Conclusions

This study investigates the undergraduate students in CS/EE to measure their perceived benefits from the experiences in service learning coursework. By the questionnaire, we explore the significance of the perceived benefits from service learning and the correlation between their professional disciplines.

The results show that the students perceive significant benefits from service learning, which is consistent with the results of [4, 5]. But the students recognize fair confidence from professional learning. Because nearly 67.24% of the subjects are first-year or sophomore-year students, the capabilities perceived from professional discipline manifest their premature background. Besides, the perceived mutual effects between service learning and professional learning are denied. However, we find that the perceived benefits from both service learning and professional learning are positively correlated to the effect of service learning (professional learning) on professional learning (service learning). The results are potentially compatible with Gallini and Moely's finding [8] that the service learning courses promoted interpersonal, community, and academic engagement, were academic challenging, and encouraged their continued study at the university (retention). So we claim that students' perceived benefits from service learning and professional confidence are correlated with their mutual effects in a mediated way. Once students perceive high benefits from service learning or professional learning, they can also reinforce the confidence in either service learning or professional discipline.

Besides, we design the classification model using naïve Bayesian networks for predicting the benefits of service learning that students perceive. By linking students' personal profiles and their feedback on service learning experiences, we may predict their attitudes (level of perceived benefits) toward service learning. Future studies are suggested to enrich the predicting variables from students' profile and internal responses. Further the classification model can be extended as a knowledge base in the counseling systems for students learning and career path.

## Figures and Tables

**Table 1:** The structure of the questionnaire.

Part 1: benefits from service learning	
(1) Personal growth	
(2) Ability to work with others	
(3) Leadership skills	
(4) Communications skills	
(5) Understanding cultural and racial differences	
(6) Social responsibility and citizenship skills	
(7) Community involvement	
(8) Applying knowledge to the “real world”	
(9) Problem analysis and critical thinking	
(10) Social self-confidence	
(11) Conflict resolution	
(12) Ability to assume personal responsibility	
(13) Development of caring relationships	
(14) Gaining the trust of others	
(15) Sensitivity to the plight of others	
(16) Workplace skills	
(17) Ability to make a difference in the community	
(18) Skills in learning from experience	
(19) Organizational skills	
(20) Connecting theory and practice	

Part 2: confidence from professional learning	
(1) Basis in CS/EE, mathematics, science, and engineering	
(2) Design/implementation of experiments, analysis, and explanation of results	
(3) Capability for using programming languages, application systems, and instruments of CS/EE	
(4) Capability for hardware and software development	
(5) Computer hardware design and computer networks	
(6) Teamwork and communication skills	
(7) Discovery, understanding, integration, and problem-solving in CS/EE	
(8) Understanding the impacts of CS/EE on environments and continuous learning	
(9) Understanding the ethical and social responsibilities of CS/EE	

Part 3: correlation between service learning and professional discipline	
(1) The capability and experience gained from service learning assist in learning the professional discipline.	
(2) The capability and experience in the professional discipline assist in the service learning coursework.	

Part 4: personal information	
Please describe your experiences in service learning in words, for example, the remarkable interactions, how your experiences from service learning assisted professional learning, or the contributions of your professional background that might help in service learning	
Gender	
Year	
Major	
Organization served	
Type(s) of services	

**Table 2:** The rotated factor matrix of service learning benefits.

Item∗	Factor			
	1	2	3	4
(13) Development of caring relationships	**0.964**	−0.208	−0.030	0.153
(16) Workplace skills	**0.894**	−0.018	0.178	−0.191
(12) Ability to assume personal responsibility	**0.893**	−0.050	0.214	−0.128
(14) Gaining the trust of others	**0.621**	0.383	−0.027	−0.100
(17) Ability to make a difference in the community	**0.560**	0.238	−0.374	0.471
(18) Skills in learning from experience	**0.542**	0.141	0.127	0.172
(9) Problem analysis and critical thinking	−0.120	**0.903**	0.012	0.158
(8) Applying knowledge to the “real world”	−0.149	**0.867**	0.030	0.176
(2) Connecting theory and practice	0.207	**0.705**	0.066	0.028
(11) Conflict resolution	0.128	**0.683**	0.212	−0.059
(10) Social self-confidence	0.011	**0.624**	0.389	−0.089
(19) Organizational skills	0.322	**0.474∗∗**	0.229	0.007
(20) Ability to work with others	0.143	0.024	**0.809**	0.030
(1) Personal growth	0.102	0.060	**0.700**	0.162
(4) Communications skills	−0.004	0.367	**0.619**	−0.017
(3) Leadership skills	−0.097	0.180	**0.500**	0.381
(5) Understanding cultural and racial differences	−0.283	0.118	0.211	**0.853**
(7) Community involvement	0.342	0.094	−0.093	**0.614**
(6) Social responsibility and citizenship skills	0.101	−0.110	0.413	**0.582**
(15) Sensitivity to the plight of others	0.366	−0.054	0.258	**0.481∗∗**

*The four factors are denominated as (1) practical skills and development, (2) critical thinking, (3) leadership and interpersonal skills, and (4) citizenship, respectively.

**Factor loading slightly below 0.5.

**Table 3:** The descriptive statistics by factor.

Item	Mean	SD	
Part 1: benefits of service learning			
Factor 1:*practical skills and development *			
(13) Development of caring relationships	5.069	1.192	
(16) Workplace skills	5.112	1.061	
(12) Ability to assume personal responsibility	5.164	1.079	
(14) Gaining the trust of others	4.897	1.211	
(17) Ability to make a difference in the community	4.603	1.179	**∗**
(18) Skills in learning from experience	5.026	1.219	
Factor 2:*critical thinking *			
(9) Problem analysis and critical thinking	4.603	1.257	**∗**
(8) Applying knowledge to the “real world”	4.552	1.301	**∗**
(20) Connecting theory and practice	4.647	1.232	
(11) Conflict resolution	4.647	1.274	
(10) Social self-confidence	4.759	1.206	
(19) Organizational skills	4.776	1.238	
Factor 3:*leadership and interpersonal skills *			
(2) Ability to work with others	5.164	1.095	
(1) Personal growth	4.983	1.209	
(4) Communications skills	5.000	1.165	
(3) Leadership skills	4.569	1.113	**∗**
Factor4:*citizenship *			
(5) Understanding cultural and racial differences	4.422	1.252	**∗∗**
(7) Community involvement	4.802	1.287	
(6) Social responsibility and citizenship skills	4.974	1.226	
(15) Sensitivity to the plight of others	4.879	1.188	
Part 2: benefits from professional learning			
(1) Basis in CS/EE, mathematics, science, and engineering	4.862	0.903	
(2) Design/implementation of experiments, analysis, and explanation of results	4.828	0.897	
(3) Capability for using programming languages, application systems, and instruments of CS/EE	4.716	0.921	∗
(4) Capability for hardware and software development	4.629	0.956	∗
(5) Computer hardware design and computer networks	4.595	0.884	∗
(6) Teamwork and communication skills	4.836	0.978	
(7) Discovery, understanding, integration, and problem-solving in CS/EE	4.672	0.921	∗
(8) Understanding the impacts of CS/EE on environments and continuous learning	4.698	0.877	∗
(9) Understanding the ethical and social responsibilities of CS/EE	4.776	0.866	∗
Part 3: correlation between service learning and professional discipline			
(1) The capability and experience gained from service learning assist in learning the professional discipline.	4.302	1.217	∗∗
(2) The capability and experience in the professional discipline assist in the service learning coursework.	4.181	1.269	∗∗
Part 4: personal background			
Gender			
Male: 86 (74.14%) Female: P 30 (25.86%)			
Year			
Freshmen: 40 (34.48%) Sophomore: 38 (32.76%) Junior: 4 (3.45%)			
Senior: 34 (29.31%)			
Major			
Computer science: 56 (48.28) Electric engineering: 59 (50.86%)			
Place served∗∗∗			
On-campus: 87 (75%) Off-campus: 56 (48.28%)			
Type(s) of services∗∗∗			
Work assistance: 68 (58.62%) Life assistance: 40 (34.48%)			
Document processing: 36 (31.03%) Escort services: 24 (20.67%)			
Schoolwork assistance: 14 (12.07%)Activities planning: 9 (7.76%)			
Others: 11 (9.48%)			

*Rejected by the *t*-test “mean ≥ 5” but accepted by hypotheses “mean ≥ 4.5”, 99% confidence.

**Rejected by the *t*-test “mean ≥ 4.5”, 99% confidence.

***Sum beyond 100% because a student may attend services in various types or places.

**Table 4:** Nodes of the Bayesian network on service learning.

Node	Description	States
*SL *	Level of perceived benefits from service learning	[1, 7]
*Gen *	Gender	{1: male, 2: female}
*Gra *	Grade	{1, 2, 3, 4+}
*Pla *	Place of service	{1: on-campus, 2: off-campus, 3: both}
*Org*1	Organization serviced: schools	{1: yes, 0: no}
*Org*2	Organization serviced: NGO	{1: yes, 0: no}
*Org*3	Organization serviced: government(s)	{1: yes, 0: no}
*Srv*1	Service type: life assistance	{1: yes, 0: no}
*Srv*2	Service type: work assistance	{1: yes, 0: no}
*Srv*3	Service type: schoolwork assistance	{1: yes, 0: no}
*Srv*4	Service type: escort services	{1: yes, 0: no}
*Srv*5	Service type: activities planning	{1: yes, 0: no}
*Srv*6	Service type: document processing	{1: yes, 0: no}
*TF*1	Term in feedback: “learning”	{1: significant, 0: not significant}
*TF*2	Term in feedback: “service”	{1: significant, 0: not significant}
*TF*3	Term in feedback: “profession”	{1: significant, 0: not significant}
*TF*4	Term in feedback: “process”	{1: significant, 0: not significant}
*TF*5	Term in feedback: “document”	{1: significant, 0: not significant}
*TF*6	Term in feedback: “capability”	{1: significant, 0: not significant}

**Table 5:** The classification accuracy of the Bayesian network.

Fold	3-state root	5-state root
1	0.8189	0.3982
2	0.7931	0.4483
3	0.6897	0.4828
4	0.8276	0.5172
5	0.8966	0.5517
Average	**0.8052**	**0.4796**
